# Soluble cytokines and chemokines in NSCLC: drivers of immune evasion and angiogenesis

**DOI:** 10.3389/fimmu.2026.1699065

**Published:** 2026-03-10

**Authors:** Liang Yang, Zhijun Fan, Zhe Wang, Dong Zhou

**Affiliations:** 1 Department of Radiotherapy, Affiliated Zhongshan Hospital of Dalian University, Liaoning, Dalian, China; 2 Department of Medical Oncology, Affiliated Zhongshan Hospital of Dalian University, Liaoning, Dalian, China

**Keywords:** angiogenesis, chemokine, cytokine, immunotherapy, metastasis, non-small cell lung cancer, PD-1, soluble mediator

## Abstract

Non-small cell lung cancer (NSCLC) remains a leading cause of cancer-related mortality worldwide. The tumor microenvironment (TME) is characterized by a dynamic network of soluble cytokines and chemokines that orchestrate immune evasion, promote angiogenesis, and facilitate metastatic dissemination. Among these, interleukins such as IL-6, IL-8, and IL-10, along with chemokine axes including CXCL12–CXCR4 and CCL21–CCR7, are critical drivers of tumor progression and resistance to immunotherapy. These mediators modulate immune cell recruitment, epithelial–mesenchymal transition, and vascular remodeling, thereby shaping tumor behavior and therapeutic response. In parallel, angiogenic factors such as VEGF, bFGF, and MMPs promote neovascularization and extracellular matrix degradation, reinforcing metastatic potential. Notably, cytokine signatures in peripheral blood are emerging as prognostic biomarkers and predictive indicators for immune checkpoint blockade efficacy, particularly PD-1 inhibitors. This review systematically summarizes the current understanding of soluble mediator-driven mechanisms in NSCLC progression, including cytokines and chemokines, providing new opportunities for biomarker-guided precision therapy and combination strategies in NSCLC.

## 1 Introduction

Lung cancer remains among the most prevalent and lethal malignancies globally, constituting the leading cause of cancer-related mortality worldwide (1). Within the tumor microenvironment (TME), elevated cytokine concentrations and dense inflammatory infiltrates are hallmarks of disease progression (2). Chronic inflammation, sustained by persistent cytokine signaling, plays a critical role in oncogenesis and tumor evolution. Interleukins and chemokines serve as central mediators of immune escape, stromal remodeling, and neovascularization (3). For instance, interleukin-6 (IL-6) activates oncogenic pathways via the JAK2–STAT3 axis, thereby fostering tumor cell survival and immune resistance (4, 5). Conversely, IL-10 impairs cytotoxic T cell function and induces immunosuppressive macrophage polarization, facilitating immune evasion (6, 7). Chemokines such as CXCL8 (IL-8) enhance neutrophil and myeloid-derived suppressor cell (MDSC) recruitment, while promoting epithelial–mesenchymal transition (EMT), a key step in metastatic dissemination (8). The CXCL12–CXCR4 and CCL21–CCR7 axes further direct tumor cell migration and stimulate lymphangiogenesis, promoting secondary tumor colonization and immune exclusion (9, 10).

Circulating cytokines not only reflect local immune dynamics but also correlate with systemic inflammation and immune surveillance status (11, 12). Notably, tumor angiogenesis has been recognized as a tightly regulated, cytokine-driven process central to metastasis (13, 14). While conventional NSCLC treatments include surgical resection, cytotoxic chemotherapy, and molecular targeted therapies, the emergence of immune checkpoint inhibitors (ICIs) has transformed the therapeutic paradigm. Temporal shifts in serum cytokine levels have demonstrated prognostic utility, particularly in predicting responses to PD-1 blockade (15). This review summarizes the soluble cytokine and chemokines-mediated mechanisms in NSCLC metastasis, systematically analyzes angiogenesis-related cytokines, and describes how peripheral cytokine signatures modulate the efficacy of PD-1–targeted immunotherapies.

## 2 Soluble mediators in NSCLC progression

### 2.1 Chemokines in NSCLC metastasis

Chemokines orchestrate leukocyte trafficking and are pivotal in modulating tumor progression, immune evasion, and metastasis in NSCLC. CXCR4, broadly expressed across tissues, promotes oncogenesis via inflammatory signaling and homeostatic disruption (16). Its ligand, CXCL12, is frequently overexpressed in metastatic niches, facilitating tumor cell intravasation and angiogenesis (17, 18). Immunohistochemical data reveal CXCR4 localization on stromal and nuclear compartments (19). Notably, Arvind et al. demonstrated that pharmacologic blockade of CXCR4 with AMD3100 in H1299-luc2 xenografts redirected metastasis from soft tissues to bone, mimicking osteolytic patterns post-intracardiac injection and offering a robust late-stage metastasis model (20). CCR7, expressed in maturing lymphocytes and dendritic cells, governs lymphoid homing via CCL19/CCL21 binding and contributes to tumor dissemination (21). PI3K pathway activation downstream of CCR7 enhances metastatic potential in head and neck cancers (22). In NSCLC, CCR7 overexpression induces ERK1/2 and Akt phosphorylation, accelerating G2/M transition through cyclin A/B1 and CDK1 upregulation (23–25). CCL21 gradients further modulate the tumor immune milieu by recruiting CCR7^+^ regulatory T cells (Tregs), thereby suppressing CD8^+^ T cell and antigen-presenting cell function. High intratumoral CCL21 levels correlate with increased Treg infiltration and poorer prognosis. Intriguingly, engineered CCL21 overexpression may under specific conditions prime dendritic cell–mediated CD8^+^ responses, highlighting its context-dependent duality.

The CCR9–CCL25 axis, frequently co-expressed in multiple malignancies, orchestrates leukocyte migration and tumor cell infiltration by activating PI3K/Akt-dependent prosurvival pathways, thereby facilitating tumor progression (26). In the context of lung cancer, CCL25 has been shown to upregulate matrix metalloproteinases MMP2 and MMP9 while suppressing TIMP1 expression, collectively promoting extracellular matrix degradation and enhancing invasiveness (27). Notably, elevated CCR9 expression correlates with increased tumor burden and regional lymph node metastasis, underscoring its clinical relevance as a prognostic biomarker (28). Parallel to this, CXCR5—the exclusive receptor for CXCL13—regulates the homing of B and T lymphocytes to secondary lymphoid organs and plays a pivotal role in the neogenesis of tumor-associated lymphoid structures (29). Singh et al. observed significantly elevated serum levels of CXCL13 in NSCLC, accompanied by increased CXCR5 expression within the peritumoral stroma, suggesting an immunologically active tumor microenvironment (30). The CXCL13–CXCR5 axis not only fosters lymphotropism but also augments metastatic potential, with pronounced activity in adenocarcinomas compared to squamous cell carcinomas, implying a subtype-specific dependency on chemokine signaling (31).

### 2.2 Interleukin modulates NSCLC immunity and metastasis

Interleukin-8 (IL-8) exerts pleiotropic effects within the tumor microenvironment of NSCLC, acting as a potent chemotactic factor for neutrophils while simultaneously promoting tumorigenic processes such as cellular proliferation, angiogenesis, and metastatic dissemination. Elevated IL-8 levels in individuals diagnosed with late-stage pulmonary carcinoma exhibit a strong correlation with enhanced lymphatic invasion and increased angiogenic activity within NSCLC (32). The interaction of IL-8 with its cognate receptors, CXCR1 and CXCR2, activates intracellular signaling cascades that stimulate NSCLC proliferation, induce endothelial sprouting, and elevate the expression of matrix-degrading enzymes such as metalloproteinases, thereby promoting basement membrane disruption and cancer cell invasion (33). In contrast, IL-10 contributes to tumor progression by modulating the apoptotic machinery and sculpting the immune microenvironment through both autocrine and paracrine loops. Notably, Filch et al. demonstrated that systemic IL-10 administration not only reprograms tumor metabolic activity but also paradoxically enhances antitumor immunity in preclinical models (34). Within NSCLC, tumor-associated macrophages (TAMs) exhibiting heightened IL-10 secretion foster an immunosuppressive Th2-dominant profile, which facilitates immune tolerance and extracellular matrix reorganization, ultimately sustaining tumor survival and expansion.

**Figure 1:**
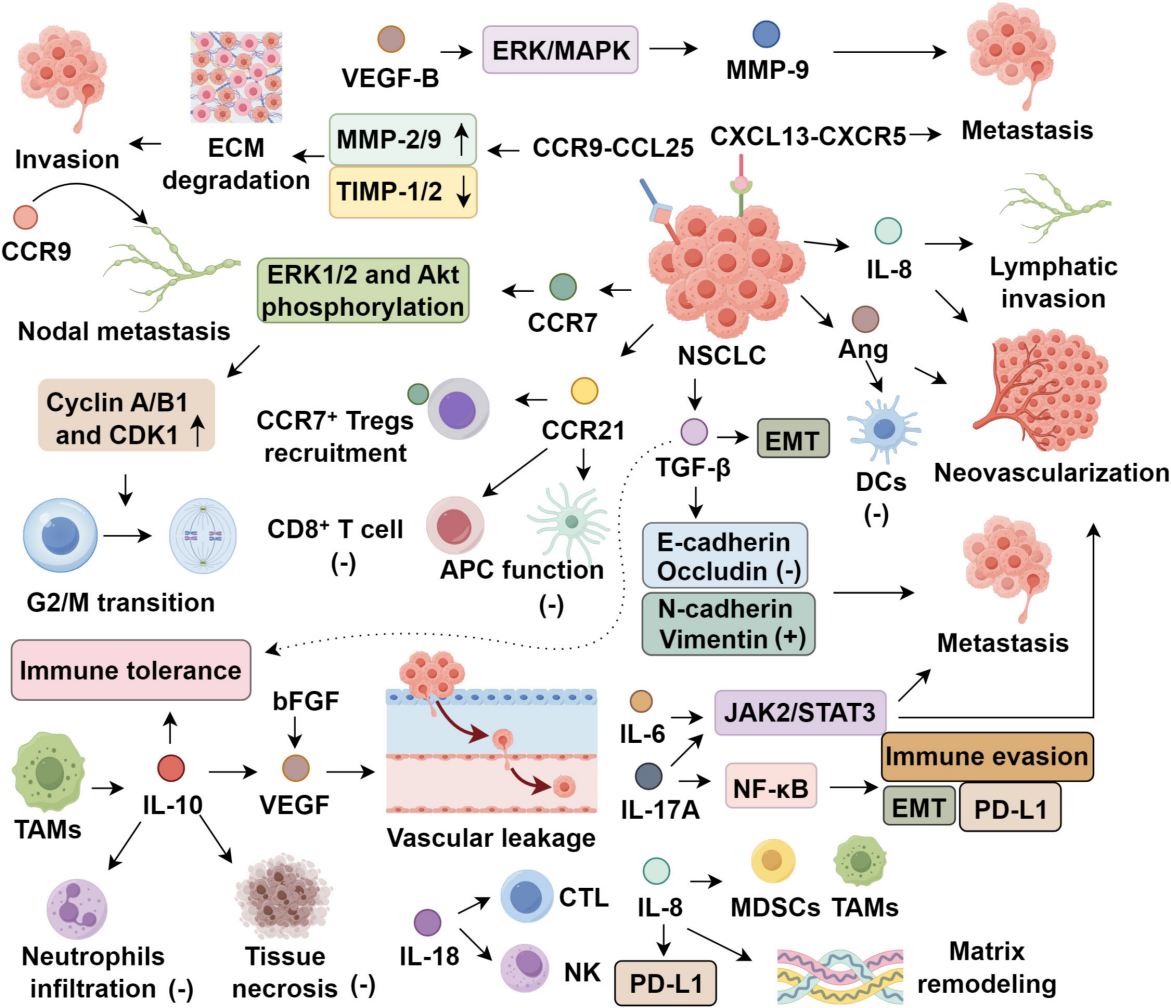
Roles of soluble cytokines and chemokines in non-small cell lung cancer.

TAMs producing IL-10 accumulate predominantly in advanced stages of NSCLC (35). IL-10 acts to suppress the infiltration of neutrophils, thereby mitigating tissue necrosis within tumor sites (36). Simultaneously, it promotes the upregulation of pro-angiogenic factors—most notably VEGF—which enhances vascular leakage and facilitates hematogenous tumor dissemination. In addition, IL-10 directly counteracts apoptotic pathways in lung cancer cells, thereby sustaining survival signals essential for the establishment and colonization of metastatic niches (37). IL-18, on the other hand, predominantly functions as an immunostimulatory cytokine by activating effector lymphocytes such as cytotoxic T cells and natural killer (NK) cells, in part by inducing major histocompatibility complex class I (MHC-I) molecule expression. Remarkably, forced IL-18 overexpression was associated with a tripling of metastatic lesions in experimental NSCLC models. Investigations by Bian C et al. (38) further confirmed IL-18’s role in potentiating interferon-γ–mediated signaling cascades and expanding the T-cell compartment, underscoring its dual role as both an immune enhancer and a modulator of tumor progression (**Figure 1**).

### 2.3 Macrophage-secreted MMP-12 and circulating MMP-9

Matrix metalloproteinase-9 (MMP-9), a gelatinase targeting basement membrane components such as gelatin and type IV–V collagens, is widely secreted across malignancies. In NSCLC, both tumor-derived and serum MMP-9 levels are markedly higher than those in benign pulmonary diseases (39), suggesting a key role in metastasis. Mechanistic studies show a feedback loop between MMP-9 and VEGF-B: VEGF-B overexpression induces MMP-9 via ERK/MAPK, while PI3K/Akt inhibition reduces VEGF-B in MMP-9–rich tumors (40), reflecting mutual angiogenic reinforcement. Clinically, MMP-9 decreases significantly after chemotherapy in advanced NSCLC, supporting its value as a treatment-responsive biomarker (39). MMP-12, primarily secreted by macrophages, degrades elastin and fibronectin and catalyzes the conversion of plasminogen to angiostatin, a potent anti-angiogenic factor (41). NSCLC tissues exhibit significantly higher MMP-12 expression than normal lung, and myeloid-specific deletion of MMP-12 disrupts hematopoietic homeostasis and exacerbates lung tumorigenesis (42, 43). Immunohistochemical analyses reveal MMP-12 localization predominantly in malignant epithelial cells, with expression levels positively correlating with advanced stage, metastatic dissemination, and recurrence, particularly within the squamous cell carcinoma subtype (44).

### 2.4 Pro-angiogenic mechanisms of bFGF and VEGF in NSCLC development

As a potent mitogenic factor, basic fibroblast growth factor (bFGF) orchestrates the proliferation of fibroblasts, epithelial cells, and vascular endothelial cells, while concurrently regulating extracellular matrix remodeling. These effects are principally mediated via fibroblast growth factor receptors (FGFRs), members of the receptor tyrosine kinase family that activate downstream angiogenic and proliferative signaling cascades (45). Heparanase synergistically interacts with bFGF, facilitating tumor invasion and dissemination through diverse mechanisms. Co-expression of bFGF and vascular endothelial growth factor (VEGF) correlates strongly with lung cancer progression and heightened microvascular density, underscoring bFGF’s pivotal role in neovascularization (46, 47). Elevated bFGF expression across the continuum from premalignant lesions to invasive and metastatic stages highlights its relevance to cancer evolution (46). The broader protein group includes various isoforms such as VEGF-A to VEGF-E, alongside platelet-derived growth factor (PDGF). Among these, VEGF-A emerges as the most potent stimulator of angiogenesis, displaying a vascular permeability capacity that surpasses that of histamine (48). VEGF-A is produced at high levels by tumor cells, stromal components, and immune infiltrates, where it drives vascular leakage and facilitates fluid accumulation, particularly in serous effusions. Importantly, malignant exudates consistently demonstrate significantly elevated VEGF-A concentrations compared to benign samples, indicating its promise as a diagnostic biomarker (49, 50). VEGF-C and VEGF-D promote the formation of lymphatic vessels by engaging VEGFR-3, thereby supporting the metastatic transit via the lymphatic system (51). When combined with positron emission tomography (PET), these biomarkers significantly enhance the accuracy of NSCLC staging (52). The prognostic utility of VEGF in surgically resected NSCLC has been statistically corroborated by Fontanini and colleagues, primarily through its impact on vascular features (53). Notably, the functional landscape of VEGF, bFGF, and other soluble mediator straddles both metastatic and angiogenic domains, creating mechanistic overlap between tumor dissemination and vascular remodeling.

## 3 Soluble mediators in NSCLC angiogenesis

### 3.1 VEGF-mediated angiogenesis in NSCLC

The formation of new vasculature within NSCLC is governed by a complex interplay between pro- and anti-angiogenic mediators, with neoplastic angiogenesis arising when pro-angiogenic cues surpass inhibitory controls (54). Tumors depend on vascular invasion and the generation of new blood conduits for metastatic competence, processes largely orchestrated by cytokine families including VEGF, angiopoietins, TGF, and TNF. Two principal modes of tumor vascularization have been delineated: sprouting angiogenesis from existing vasculature, and aberrant mobilization of endothelial progenitors to establish novel vessels, both of which are operative during lung tumorigenesis. VEGF engagement with endothelial receptor tyrosine kinases activates signaling cascades that promote vascular permeability, tubular morphogenesis, and endothelial proliferation—key events in NSCLC metastatic facilitation. These VEGFR-mediated processes support endothelial integrity and motility (55). circulating VEGF levels are significantly elevated in NSCLC patients compared to individuals with benign pulmonary lesions, underscoring its role as a marker for pathological neovascular remodeling (56). Pro-angiogenic stimuli activate endothelium by initiating intricate intracellular programs. Within NSCLC, angiopoietins (Angs) act as essential regulators. The Ang family comprises four isoforms (Ang-1 through Ang-4), which differentially modulate the Tie2 receptor: Ang-1 binding stabilizes endothelial networks, whereas Ang-2 binding disrupts vascular cohesion. Unlike the ubiquitously expressed Ang-1, Ang-2 is predominantly found in endothelial cells and is upregulated under pathological conditions. By antagonizing Ang-1, Ang-2 fosters vascular adaptability and facilitates sprouting (57, 58). While quiescent vasculature exhibits minimal Ang-2 expression, its abundance increases markedly under inflammatory or angiogenic stimuli. During the initial phase of NSCLC angiogenesis formation, Ang-2 may paradoxically hinder tumor progression by inducing vessel regression (59), while concurrently priming endothelial cells for subsequent activation. VEGF overexpression in this primed environment heightens endothelial responsiveness, culminating in the disorganized angiogenesis characteristic of malignant growth. Beyond its vascular effects, Ang-2 also contributes to immune escape by suppressing the maturation and antigen-presenting capacity of dendritic cells. This impairment of DC function weakens the initiation of adaptive immune responses, thereby promoting immune tolerance within the tumor microenvironment (60). Such dual functionality positions Ang-2 as a key molecular bridge linking aberrant angiogenesis with immunosuppressive reprogramming in NSCLC.

### 3.2 TGF-β and TNF -mediated angiogenesis in NSCLC

Angiogenesis in NSCLC is orchestrated through a cascade involving VEGF-induced vascular permeability and Ang-2-mediated destabilization, wherein TGF-β functions as a pivotal enhancer. This multifunctional cytokine governs diverse biological processes such as cellular proliferation, programmed cell death, morphogenesis, lineage commitment, stem cell niche regulation, motility, and immune modulation (61). The role of TGF−β in tumorigenesis is profoundly context−dependent: it functions primarily as a growth−suppressive factor during early tumor stages, but transitions to a potent promoter of invasion and metastasis in advanced NSCLC (62). During initial oncogenic transformation, TGF-β enforces growth arrest and apoptosis; however, in progressive disease, it facilitates metastasis via both Smad-dependent and Smad-independent EMT signaling (63). EMT confers invasive capabilities through suppression of epithelial markers (E-cadherin, occludin) and upregulation of mesenchymal proteins (N-cadherin, vimentin). TGF-β promotes EMT by initiating Sp1-dependent phosphorylation of Smad2/3, enabling their nuclear localization and downstream transcriptional activation. Furthermore, studies by Benckert et al. (64) demonstrated that TGF−β drives the upregulation of VEGF expression, highlighting a synergistic interplay between these pathways in promoting angiogenesis and NSCLC progression. Beyond its role in angiogenesis and EMT, TGF-β also exerts profound immunosuppressive effects by hindering CD8^+^ T cell infiltration into the tumor microenvironment. Mechanistically, TGF-β signaling restricts the migration and cytotoxic function of effector T cells, thereby contributing to immune exclusion and resistance to immune checkpoint therapies targeting the PD-1/PD-L1 axis. Studies have demonstrated that blockade of TGF-β can restore CD8^+^ T cell infiltration and sensitize tumors to anti–PD-1 therapy, suggesting a key role of TGF-β in orchestrating immune evasion and therapy resistance (65). These findings highlight the importance of TGF-β as a dual modulator of both tumor angiogenesis and immune suppression in NSCLC.

The TNF cytokine superfamily comprises 19 structurally distinct ligands and 29 corresponding receptors (66). Prominent members, including TNF−α, TRAIL (TNF−related apoptosis−inducing ligand), and FasL (Fas ligand), exhibit differential receptor specificity; notably, TNF−α binds exclusively to TNFR1 and TNFR2. TNF-α is initially expressed as a membrane-anchored precursor (memTNF), which undergoes proteolytic cleavage to release its soluble form (sTNF) (67). The pleiotropic biological actions of TNF−α are transduced through these two distinct receptors. TNFR1, which is constitutively and broadly expressed across most cell types, predominantly initiates apoptotic and pro−inflammatory signaling cascades. In contrast, TNFR2 displays a more restricted expression pattern and is principally linked to pro−survival and immunoregulatory pathways. TNFR2 plays a particularly critical role in adaptive immunity by modulating the expansion, survival, and functional differentiation of CD4^+^ and CD8^+^ T cells during host defense responses (68). This receptor dualism establishes a functional contrast: TNFR1 primarily drives inflammatory responses, whereas TNFR2 contributes to the maintenance of immune homeostasis (64). Within the characteristically pro−inflammatory tumor microenvironment of non−small cell lung cancer (NSCLC), TNF−α may thus operate in a manner analogous to classical growth factors, potentiating oncogenic signaling networks and thereby exacerbating tumor progression and aggressiveness.

## 4 Soluble inflammatory cytokines shaping PD-1 inhibitor efficacy in NSCLC

IL-6, a pro-inflammatory cytokine, promotes tumor proliferation, angiogenesis, and immune evasion via sustained JAK2/STAT3 activation, creating a self-reinforcing inflammatory loop (69–72). Clinically, elevated IL-6 correlates with tumor burden and cachexia (73). Importantly, decreased IL-6 levels post–PD-1 therapy has been associated with prolonged survival and improved clinical responses (74–76). Similarly, IL-8 exerts pro-inflammatory effects by stimulating CXCR1/2 on endothelial and immune cells, facilitating MDSC and TAM recruitment, PD-L1 expression via STAT3/mTOR, and matrix remodeling (77–79). Baseline and on-treatment elevations in IL-8 are negatively correlated with PD-1 therapy outcomes, making IL-8 a promising prognostic biomarker (80–83). IL-17A, another key pro-inflammatory cytokine, activates JAK2/STAT3 and NF-κB pathways (84–86), enhancing PD-L1 expression and promoting EMT and immune evasion. Elevated IL-17A serum levels have been observed in NSCLC patients with COPD and are associated with immune-related pneumonitis and diminished response to PD-1 blockade (87–90).

Dual-role cytokines include TNF-α and IFN-γ. TNF-α exerts context-dependent effects, activating NF-κB pathways through TNFR1 (pro-inflammatory) and TNFR2 (immune-regulatory) (68). In NSCLC, TNF-α may either amplify antitumor immunity or foster tumor progression depending on its receptor context and local cytokine milieu. Anti–TNF-α therapy (infliximab) is under investigation for its potential to enhance PD-1 efficacy (91), but conflicting findings on TNF-α levels and treatment outcomes warrant further study (92, 93). IFN-γ, a critical effector of adaptive immunity, promotes tumor antigen presentation and T cell cytotoxicity through JAK-STAT and PI3K-Akt pathways (94). Chronic IFN-γ exposure can exert selective pressure on tumor cells, favoring the survival of PD-L1–high phenotypes that are more resistant to cytotoxic lymphocytes. Moreover, IFN-γ signaling has been shown to upregulate immunosuppressive enzymes such as indoleamine 2,3-dioxygenase (IDO1), which depletes tryptophan and dampens T cell proliferation and effector function. These mechanisms collectively contribute to an immunosuppressive tumor microenvironment and acquired resistance to immune checkpoint inhibitors (ICIs). Impaired IFN-γ signaling is implicated in resistance to PD-1 blockade (95). Pre-treatment elevations or therapy-induced increases in IFN-γ have been associated with favorable survival metrics in NSCLC (96), though data from gastrointestinal malignancies and some NSCLC cohorts remain inconsistent (97–99). Thus, longitudinal monitoring of IFN-γ dynamics may serve as a stratification tool for PD-1 therapy response (**Supplementary Table 1**).

## 5 Conclusion

Soluble cytokines and chemokines constitute a highly dynamic signaling network that orchestrates the immunological and vascular landscape of NSCLC. Acting at the intersection of tumor progression, angiogenesis, and immune evasion, mediators such as IL-6, IL-8, IL-10, VEGF, bFGF, and the CXCL12–CXCR4 and CCL21–CCR7 axes exert profound influence on tumor growth, epithelial–mesenchymal transition, and metastatic dissemination. Their roles in modulating immune cell trafficking, promoting stromal remodeling, and altering the vascular niche highlight their pleiotropic functions in shaping both local tumor microenvironmental conditions and systemic immune responses. Moreover, elevated levels of circulating soluble mediators, such as VEGF-A and MMP-9, correlate with disease burden and provide valuable prognostic and diagnostic insights.

Importantly, these soluble factors also act as predictive biomarkers for immune checkpoint blockade (ICB) efficacy, particularly for PD-1/PD-L1 inhibitors. Cytokine profiles such as IL-6, IL-8, IL-17A, TNF-α, and IFN-γ influence treatment responsiveness by modulating immune cell activation, antigen presentation, and immune checkpoint expression. Understanding the dual roles of certain cytokines, such as IL-10 and TNF-α, as both immunosuppressive and immunostimulatory agents remains crucial for optimizing therapeutic strategies. Targeting these soluble mediators, either directly or in combination with ICBs, offers a promising avenue to overcome resistance and improve outcomes. Future research should prioritize longitudinal cytokine monitoring and integrative biomarker panels to refine precision immunotherapy and identify context-specific vulnerabilities in NSCLC. This integrated framework may ultimately inform the development of combination regimens that target both immune evasion and angiogenic remodeling to enhance antitumor efficacy.
